# A comprehensive investigation of the radiation isocentre spatial variability in linear accelerators: implications for commissioning, QA, and clinical protocols

**DOI:** 10.1007/s13246-025-01637-8

**Published:** 2025-09-10

**Authors:** Zhen Hui Chen, Hans Lynggaard Riis, Rohen White, Thomas Milan, Pejman Rowshanfarzad

**Affiliations:** 1 School of Physics, Mathematics and Computing, The University of Western Australia, Perth, WA Australia; 2 Department of Radiation Oncology, Sir Charles Gairdner Hospital, Nedlands, WA Australia; 3 Department of Oncology, Odense University Hospital, Odense, Denmark; 4 Department of Clinical Research, University of Southern Denmark, Odense, Denmark; 5 Centre for Advanced Technologies in Cancer Research (CATCR), Perth, WA Australia; 6 Crown Princess Mary Cancer Centre, Westmead Hospital, 166-174 Hawkesbury Rd, Westmead, NSW 2145 Australia

**Keywords:** Radiation isocentre, Treatment planning, Linac QA, Commissioning, Stereotactic radiosurgery

## Abstract

Stereotactic ablative radiation therapy and stereotactic radiosurgery (SRS) are modern high precision techniques used for cancer management. Hence, evaluating potential clinical impacts of mischaracterisation of the radiation isocentre (RI) variability at different gantry and collimator angles during commissioning and quality assurance (QA) helps to ensure the robustness of treatment delivery and successful outcomes. This was done using signal edge detection techniques on flattened and flattening filter free (FFF) fields generated by megavoltage (MV) treatment beams. A total of 22,815 MV images of 4 × 16 cm^2^, were captured using an iViewGT™ Perkin Elmer a-Si Electronic Portal Imaging Device at 13 different gantry angles, paired with 13 collimator angles each in 30° intervals. 6 MV, 6 MV FFF, and 18 MV photon treatment modes were used across 8 linacs. The Starshot images were subsequently analysed using custom in-house signal detection software developed in Python. The mean RI variabilities across the entire treatment arc were approximately 0.58 mm, 0.66 mm, and 0.58 mm, for beam energies of 6 MV, 6 MV FFF, and 18 MV respectively, with corresponding standard deviations of 0.18 mm, 0.21 mm, and 0.19 mm, with maximum variabilities occurring at 300°, 0°, and 240° of magnitudes 0.62 mm, 0.71 mm, and 0.62 mm, respectively. Correspondingly, the standard deviations were 0.20 mm, 0.23 mm, and 0.17 mm. The resulting RI variabilities, applied to SRS QA parameters, may breach the 1 mm tolerance. In some clinical scenarios, this may impact treatment plan acceptance with treatment coverage goals outside of acceptable limits. We recommend enhancing commissioning and QA protocols by combining Starshot and Winston–Lutz-style tests to assess the positional deviation of the apparent radiation isocentre from the mechanical isocentre, and the radiation isocentre’s maximal variability across a larger range of intermediate angles. This approach helps define reference angles and tailor QA frequency based on individual linac behaviour.

## Introduction

The primary goal of radiation therapy is to deliver highly conformal dose distributions to targets while minimising irradiation of surrounding healthy tissue in patients, to achieve improved outcomes and survivability [1–11]. With recent technological advancements in radiotherapy treatment devices, linacs have achieved higher mechanical precision and stability, reaching sub-millimetre levels. With the advent of SRS and Stereotactic ablative radiation therapy (SABR), there is an increased demand for highly accurate and efficient quality control methods [5, 12–18]. These methods are essential to ensure that the linacs can deliver the plans with the required accuracy and precision [19].

Additionally, there are no predictive models currently available that can integrate these variations into the TPS. The indications and demand for stereotactic radiotherapy treatment (SRT) is increasing, and as such, the significance and necessity of more accurately characterising the various components of the radiation isocentres becomes increasingly apparent.

Szalkowski et al. [20] describes the development of an in-house processing method, allowing users to conduct their own QA tests on any film or electronic portal imaging device (EPID) acquired images [21–24]. Commercially available software facilitates this process by identifying the RI, achieved through an analysis of the composite superposition of spokes within the Starshot image.

Their findings indicated that when the film was irradiated from opposing angles, the deviations from the true centre offset each other, resulting in minimal variations in the RI. However, it was observed that the variability differed for every 180° collimator angle, suggesting the potential for detecting lower variations. The EPID analysis involved segmenting a ring of sampled pixels from the Starshot image into two sets using a line perpendicular to the collimator angle specified within the DICOM file. Subsequently, a Gaussian filter was applied to both data sets to determine the centreline of the beam. To minimise any fluctuations during the determination of the “true” centreline using this ring technique, five distinct ring radii were utilised.

Drawing from Depuydt et al. [25], which explored artificial Starshot tests as a means to further enhance the performance of film digitisation and computer analysis, an analytical solution was applied to address the smallest intersecting circle problem associated with the Starshot test, in contrast to the gradient optimisation approach used. Subsequently, artificial Starshots with a known radiation isocentre were used to quantify the systematic errors introduced during the digitisation and computerised analytic process of the film. The technique yielded an estimated uncertainty of 0.04 mm for radiographic film and 0.06 mm for radiochromic film, demonstrating a comparable level of performance.

In this work, the radiation isocentre variability was characterised over various gantry and collimator angles in degrees, bringing together different definitions into a comprehensive framework for understanding and informing on the impact of the radiation isocentre during treatment planning and QA procedures. This work provides the necessary definitions and data required to understand the gaps that may be present in current commissioning and QA practices, and is intended to enable more informed decision-making during QA and treatment planning procedures.

## Methods

### Accelerators and EPIDs

The data collection involved four Elekta Synergy linacs, two Elekta Versa HD linacs, and two Philips SL 20 units, with measurements conducted using the iViewGT™ Perkin Elmer a-Si EPIDs. The detector array featured an active area of 41 × 41 cm^2^ with a resolution of 1024 × 1024 pixel, resulting in a pixel size of 0.4 × 0.4 mm^2^. Measurements were obtained at thirty-degree intervals, starting from zero, for both gantry and collimator rotations, culminating in a total of 13 angles per assessment. Consequently, each linac, per energy mode, generated 169 images for each treatment arc. Notably, linacs 3, 7, and 8 were used for SRS/SABR, which explains the acquisition of the 6 MV FFF energy mode. A summary of LINAC models, beam energies, and MLC models is given in Table 1.

**Table 1:** The list of linacs used in this and their respective MLC models

Linac	Nickname	Energy	MLC model
Elekta Synergy	Linac 1	6 MV, 18 MV	MLCi
Philips SL 20	Linac 2	6 MV, 18 MV	MLCi
Elekta Synergy	Linac 3	6 MV, 6 MV FFF, 18 MV	MLC160
Philips SL 20	Linac 4	6 MV, 18 MV	MLCi
Elekta Synergy	Linac 5	6 MV, 18 MV	MLCi2
Elekta Synergy	Linac 6	6 MV, 18 MV	MLCi2
Elekta Versa HD	Linac 7	6 MV, 6 MV FFF, 18 MV	MLC160
Elekta Versa HD	Linac 8	6 MV, 6 MV FFF, 18 MV	MLC160

### Gantry and collimator angle data

**Fig. 1:**
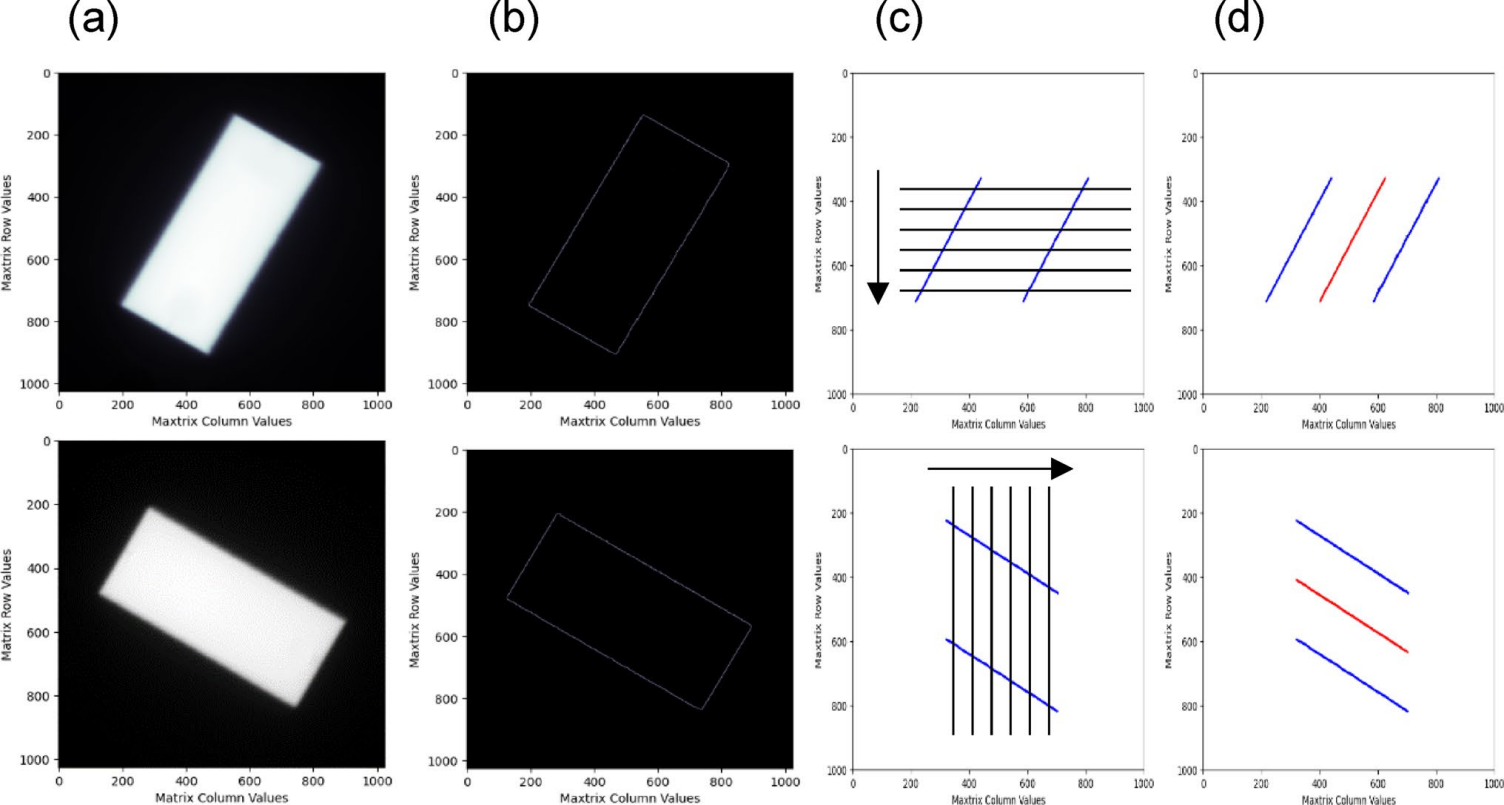
From left to right: **a** Raw DICOM Starshot field; **b** Canny edge detected pixel positions; **c** Vertical and horizontal scanning for Starshot collimator angle determination; and **d** CAX determined through linear regression curve fitting across the mean subpixel positions

**Fig. 2:**
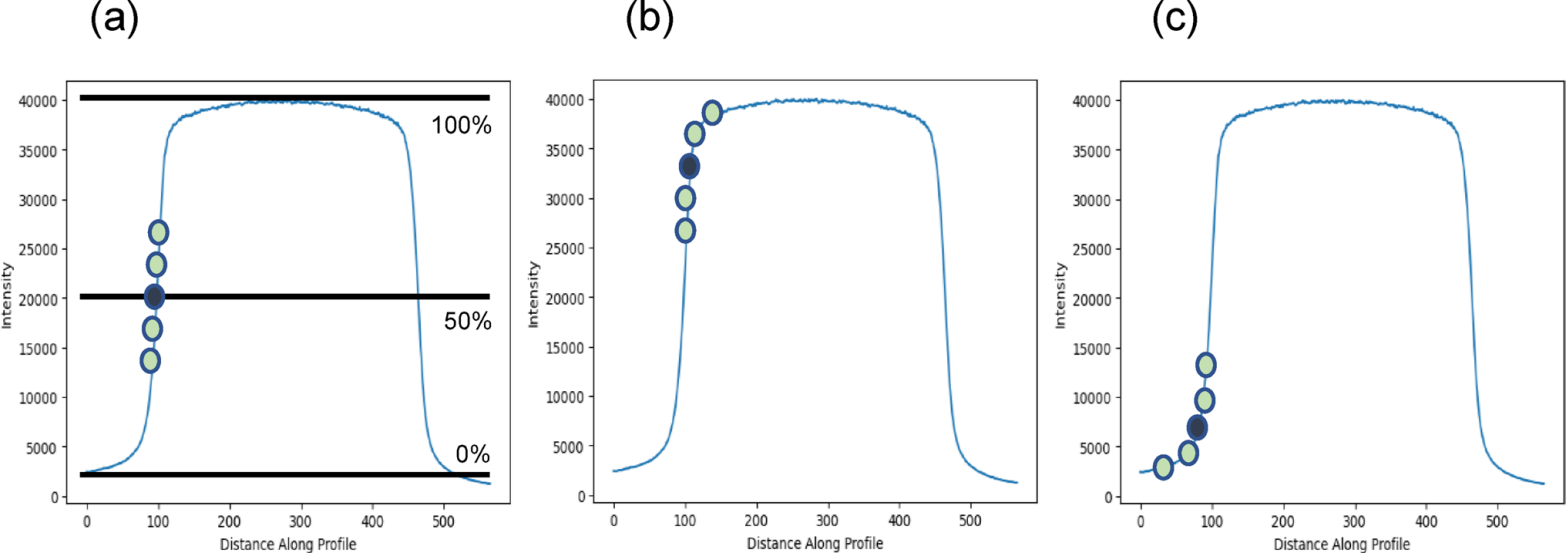
From left to right: **a** subpixel position taken at 50% intensity; **b** possibility one if subpixel position was not to be taken at 50% intensity; and **c** possibility two if subpixel position was not to be taken at 50% intensity

The Figs. 1 and 2 below illustrate the various stages of refinement as the beam central axis (CAX) was extracted using the method shown in this study.

The CAX extraction process, guided by the developed algorithm, follows Fig. 1a–c:

(i) The given set of DICOM Starshot images at different gantry and collimator angles were processed and loaded into the in-house Python software. Pixel offsets were applied, along with a median filter for noise reduction. The images were subsequently indexed for proper identification throughout the extraction procedure.
(ii) The canny edge detection approach [26] was employed to initially screen and identify the approximate pixel positions of the edges in each image. Subsequently, the detected pixels were grouped according to field alignment. If the fields exhibited a minimum-to-maximum vertical pixel positions length (column range) greater than their minimum-to-maximum horizontal pixel positions width (row range), the image was classified as a vertical scan image. Conversely, if the reverse condition was met, the image was classified as a horizontal scan image.
(iii) The vertical and horizontal classification dictate the directions in which the image profiles are extracted. In instances of vertical classification, the maximum and minimum column pixel values per row are considered, ensuring a one-to-one pairing when the image profiles are taken. Conversely, horizontally classified images involve the collection of maximum and minimum row pixel values per column. The profiles for each classification were taken at 70% in-field to remove potential distortions from peripheral noise, thereby maintaining the integrity of the linear regression fit used to determine the CAX. Systematic scanning of the profiles across the entire range of available pixels facilitated the identification of the 50% in-tensity point at both the left and right shoulders of the profile. These points served as the definitive reference positions for the edge pixel location, with one neighbouring pixel of lower intensity and two of higher intensities utilized for fitting a cubic regression. The resulting cubic function, normalized to 50% intensity, represented the subpixel position of the edge. This method was repeated for both shoulders and for each scan profile, enabling the achievement of subpixel accuracy. The subpixel value pairs were subsequently indexed.
(iv) The mean position of each subpixel value pair was determined via simple averaging, and the resulting set of mean values was fitted using a linear regression model. The resulting function was then plotted, representing the CAX of a given DICOM Starshot image. The angle of the function against the y-axis was determined by solving the arctangent function using two points found on the function. All combinations of the possible CAX configurations were then generated using the binomial coefficient, and Heron’s formula was applied to find both the incircle radius and the incentre position. Both these values are indexed to each set of images denoted by their respective gantry and collimator angles for reference during analysis. See Fig. 8. for additional information.

Please refer to “Appendix 1” for the pseudocode as reference.

### Radiation isocentre extraction

Zhang et al. [27] describe the radiation isocentre as the centre of the smallest insphere intersecting planes formed by the X-ray source and collimation centre at various gantry angles. Due to practical limitations, only a subset of these planes were sampled via gantry and collimator rotation, producing 2D projections on the EPID. The insphere is approximated in 2D by its incircle. This study uses the binomial coefficient to generate all possible triangles from Starshot line intersections and applies Heron’s [28] formula to calculate the incircle’s size and centre, serving as a proxy for radiation isocentre variability and position.

### Statistical distributions tests

Different statistical distributions tests are employed based on the characteristics of the data and assumptions underlying the required analyses. For instance, many parametric tests rely on the assumption of normality. Departure from this assumption has implications that alternative non-parametric tests may be more appropriate. Understanding the nature of the dataset, such as its distribution and underlying structure, allows for the accurate selection of statistical methods, ensuring robustness in the analysis of the data, and reliability of the results. This allows for more confident interpretations and informed decision making.

### Shapiro–Wilk test

The Shapiro–Wilk test [29] serves as a statistical tool for evaluating whether a dataset adheres to a normal distribution, a critical step in validating the normality assumption in various statistical examinations. This test computes a test statistic denoted as “W”, which quantifies the concordance between the observed order of data and the expected order assuming a normal distribution. When W approximates 1, it suggests that the data follows a normal distribution, and the null hypothesis of normality remains unchallenged.

Conversely, if W significantly deviates from 1, it signifies that the data strays from a normal distribution, leading to the rejection of the null hypothesis. The Shapiro–Wilk test proves particularly valuable for smaller sample sizes and is readily accessible within statistical software, playing a vital role in verifying that data complies with the normality assumption in parametric analyses.

### Kruskal Wallis test

The Kruskal–Wallis test [30] is a non-parametric statistical test designed to evaluate whether there are statistically significant differences among three or more independent groups. It serves as an extension of the Mann–Whitney “U” test [31] to accommodate multiple groups, making it particularly useful when the assumptions of parametric tests, such as ANOVA, are not met. The test is applied to assess variations in the medians of these groups and is most advantageous when working with non-normally distributed or ordinal data.

To compute the Kruskal–Wallis test statistic, data points are ranked across all groups, and group sums of ranks are calculated. The test results in a *p* value that, when compared to a chosen significance level, indicates whether significant differences exist among the groups. Post hoc tests are often employed to identify which specific pairs of groups exhibit differences after the Kruskal–Wallis test.

### Dunn’s test

The Dunn’s test [32, 33], a non-parametric statistical method, is employed for pairwise comparisons between groups following an omnibus non-parametric test, in our case, the Kruskal–Wallis test, which indicates significant differences among groups. It aids in pinpointing specific pairs of groups that exhibit statistically significant differences in their distributions. The test calculates test statistics (z-scores) for each pairwise comparison and derives *p* values, enabling the identification of significant group differences.

To control the risk of Type I errors in multiple comparisons, the *p* values are often adjusted using methods like Bonferroni or Benjamini-Hochberg [34, 35]. This test is particularly valuable when dealing with non-normally distributed data and when the assumption of homogeneity of variances is not met, providing a means to explore group differences in greater detail while maintaining rigorous statistical control.

The Bonferroni method was selected because of its stringent criteria for preventing Type I errors. In this instance, the primary concern is minimising false positives due to the strict size tolerance requirements of the radiation isocentre, making it the more suitable choice.

### Radiation isocentre variability calculations on brain SRS single and multi-lesion plans in TPS

To assess the potential clinical impact of RI variability, we retrieved the first two brain SRS treatment plans from the hospital Eclipse TPS repository that fulfilled pre-specified criteria of one single and one multi-lesion brain SRS, without previous radiation. The single lesion plan was of a 64-year-old female prescribed 12.5 Gy for a vestibular schwannoma. The multi-lesion SRS plan was of a 48-year-old female with four brain metastases, each prescribed to 18 Gy using a mono-isocentric technique. The prescribed dose was to the isodose line covering the PTV if the plan adequately met organs at risk (OAR) doses, conformity index, gradient indices, maximum dose and other departmental SRS criteria. If all criteria were not met, compromise was at the discretion of the treating clinician. Dose distributions were reproduced following various simulated shifts in radiation isocentre estimated by our model. Key dosimetric criteria encompassing target dose goals and OAR constraints from the brain SRS treatment protocol was then compared.

The single and multi-lesion brain SRS plans with the simulated shifts in the radiation isocentre were calculated using the Eclipse TPS. The photon dose algorithm calculation model utilized for plan evaluation was version AXB_16.1_1, 0.5 of the Acuros External Beam Version 16.1.0 beam model. Plans were optimised using the PO_1610 Photon Optimizer Version 16.1.0 model. The calculation grid was set to 0.125 cm, with dose calculation performed in medium, incorporating heterogeneity correction. Fields were normalized to 100% of the isocentre for photon volume dose calculations. Optimisation parameters included inhomogeneity correction, air cavity correction, high-resolution dose calculation, and high aperture shape controller setting. GPU utilisation was not employed during optimisation. Clinical goals were evaluated using the DVH Estimation Algorithm Version 16.1.0. The Portal Dose Image Prediction (Version 15.6.03) calculation model was employed for portal projections.

## Results

The test statistics and *p* values of the Shapiro–Wilk test across different energy levels are presented in Table 2. Table 3 displays the Kruskal–Wallis test statistic results and corresponding *p* values following the Shapiro–Wilk test. Table 4 provides the results of the final post-hoc Dunn’s test, illustrating the combinations of different energies and their corresponding statistical distinctions.

**Table 2:** Results of the Shapiro–Wilk test for the radiation isocentre sizes at 6 MV, 6 MV FFF, and 18 MV energies

Energy	Test statistic	*p* value
6 MV	0.98	1.2 × 10^−7^
6 MV FFF	0.96	9.65 × 10^−11^
18 MV	0.98	4.42 × 10^−8^

**Table 3:** Results of the Kruskal–Wallis test when comparing the results of the radiation isocentre sizes at 6 MV, 6 MV FFF, and 18 MV energies

Test statistic	*p* value
44.41	2.27 × 10^−10^

**Table 4:** Post hoc results of the Dunn’s test when comparing the Kruskal–Wallis results of the radiation isocentre sizes at 6 MV, 6 MV FFF, and 18 MV energies

	6 MV	6 MV FFF	18 MV
6 MV	1.00 × 10^0^	6.43 × 10^−8^	1.00 × 10^0^
6 MV FFF	6.43 × 10^−8^	1.00 × 10^0^	8.64 × 10^−9^
18 MV	1.00 × 10^0^	8.64 × 10^−9^	1.00 × 10^0^

**Table 5:** Summary of the number of image acquisitions by beam energy and modality, taken at 13 gantry and collimator angles (169 image sets each)

Energies	6 MV	6 MV FFF	18 MV
Linacs	Acquisition count	Acquisition count	Acquisition count
Linac 1	6	NIL	6
Linac 2	6	NIL	4
Linac 3	14	35	14
Linac 4	3	NIL	3
Linac 5	3	NIL	3
Linac 6	3	NIL	3
Linac 7	5	5	5
Linac 8	5	5	5
Total images	7605	7605	7605

A total of 22,815 Starshot images were collected, with only linacs 3, 7, and 8 delivering the 6 MV FFF modality; others had no acquisitions for this energy

**Table 6:** Summary of the clinical goals and the dose deviations from the original plan when 0.07 cm shifts were applied in the X, Y and Z directions as proxy for the mischaracterisation of radiation isocentre variability at various gantry and collimator angles during treatment for a single-lesion brain SRS plan

Structure	Clinical goal	Plan (Gy)	X		Y		Z		Minimum value (Gy)	Minimum change (%)	Maximum value (Gy)	Maximum change (%)
			+ 0.07 cm (Gy)	− 0.07 cm (Gy)	+ 0.07 cm (Gy)	− 0.07 cm (Gy)	+ 0.07 cm (Gy)	− 0.07 cm (Gy)				
PTV_2 mm	D98% ≥ 12.50 Gy	12.50	12.36	11.58	12.01	11.99	12.09	11.98	11.58	− 7.36	12.36	− 1.12
PTV_2 mm	Max < 16.63 Gy	15.99	15.84	16.03	16.00	16.02	15.90	16.03	15.84	− 0.94	16.03	0.25
GTV	D100% ≥ 12.50 Gy	14.18	13.98	14.18	14.06	14.00	13.80	13.99	13.80	− 2.68	14.18	0.00
PTV_1 mm	D 98.0% ≥ 12.50 Gy	13.92	13.73	13.82	13.68	13.67	13.53	13.59	13.53	− 2.80	13.82	− 0.72
PTV_1 mm	Dmax < 16.63 Gy	15.99	15.84	16.03	16.00	16.02	15.90	16.03	15.84	− 0.94	16.03	0.25
Brain stem	Max < 12 Gy	9.04	10.65	7.56	9.43	8.40	10.17	8.33	7.56	− 16.37	10.65	17.81
Left eye	Max < 4 Gy	0.12	0.12	0.12	0.12	0.12	0.14	0.10	0.10	− 16.67	0.14	16.67
Left lens	Max < 4 Gy	0.04	0.04	0.04	0.04	0.04	0.04	0.04	0.04	0.00	0.04	0.00
Left optic nerve	Max < 4 Gy	0.09	0.09	0.08	0.09	0.09	0.09	0.08	0.08	− 11.11	0.09	0.00
Optic chiasm	Max < 4 Gy	0.21	0.25	0.21	0.21	0.21	0.22	0.21	0.21	0.00	0.25	19.05
Right eye	Max < 4 Gy	0.18	0.17	0.19	0.19	0.19	0.23	0.17	0.17	− 5.56	0.23	27.78
Right lens	Max < 4 Gy	0.08	0.08	0.08	0.08	0.08	0.09	0.08	0.08	0.00	0.09	12.50
Right optic nerve	Max < 4 Gy	0.27	0.27	0.23	0.24	0.27	0.25	0.29	0.23	− 14.81	0.29	7.41

Calculations were made using the Eclipse TPS

**Table 7:** Summary of the clinical goals and the dose deviations from the original plan when 0.07 cm shifts were applied in the X, Y and Z directions as proxy for the mischaracterisation of radiation isocentre variability at various gantry and collimator angles during treatment for a multi-lesion brain SRS plan

Structure	Clinical goal	Plan (Gy)	X		Y		Z		Minimum value (Gy)	Minimum change (%)	Maximum value (Gy)	Maximum change (%)
			+ 0.07 cm (Gy)	− 0.07 cm (Gy)	+ 0.07 cm (Gy)	− 0.07 cm (Gy)	+ 0.07 cm (Gy)	− 0.07 cm (Gy)				
GTV1	D100% ≥ 18 Gy	20.49	20.14	19.98	20.04	19.97	20.31	19.86	19.86	− 3.07	20.31	− 0.88
GTV2	D100% ≥ 18 Gy	20.99	20.61	20.61	20.86	20.70	20.54	20.28	20.28	− 3.38	20.86	− 0.62
GTV3	D100% ≥ 18 Gy	21.31	20.43	20.59	20.62	20.74	20.73	20.66	20.43	− 4.13	20.74	− 2.67
GTV4	D100% ≥ 18 Gy	21.22	20.44	20.85	20.59	20.71	20.64	20.75	20.44	− 3.68	20.85	− 1.74
PTV1_1 mm	D98% ≥ 18 Gy	20.22	19.7	19.85	19.81	19.84	19.95	19.64	19.64	− 2.87	19.95	− 1.34
PTV2_1 mm	D98% ≥ 18 Gy	20.27	19.91	19.82	19.92	19.86	19.74	19.65	19.65	− 3.06	19.92	− 1.73
PTV3_1 mm	D98% ≥ 18 Gy	20.74	20.21	20.14	19.99	20.20	19.94	19.68	19.68	− 5.11	20.21	− 2.56
PTV4_1 mm	D98% ≥ 18 Gy	20.57	20.11	20.19	20.04	20.23	19.86	19.93	19.86	− 3.45	20.23	− 1.65
PTV1_2 mm	D98% ≥ 18 Gy	18.10	17.44	17.61	17.67	17.65	17.66	17.51	17.44	− 3.65	17.67	− 2.38
PTV1_2 mm	Max ≤ 23.94 Gy	23.80	23.88	23.76	23.84	23.76	23.84	23.79	23.76	− 0.17	23.88	0.34
PTV2_2 mm	D98% ≥ 18 Gy	18.03	17.33	17.18	17.61	17.35	17.41	17.13	17.13	− 4.99	17.61	− 2.33
PTV2_2 mm	Max ≤ 23.94 Gy	23.21	23.24	23.13	23.27	23.16	23.24	23.16	23.13	− 0.34	23.27	0.26
PTV3_2 mm	D98% ≥ 18 Gy	18.00	17.32	16.97	16.94	17.10	17.02	16.82	16.82	− 6.56	17.32	− 3.78
PTV3_2 mm	Max ≤ 23.94 Gy	23.34	23.26	23.37	23.37	23.30	23.29	23.21	23.21	− 0.56	23.37	0.13
PTV4_2 mm	D98% ≥ 18 Gy	18.02	17.26	17.09	16.97	16.89	17.15	17.05	16.89	− 6.27	17.26	− 4.22
PTV4_2 mm	Max ≤ 23.94 Gy	23.56	23.45	23.55	23.53	23.54	23.51	23.43	23.43	− 0.55	23.55	− 0.04
Brain stem	V10 Gy < 0.5 cc	0.00	0.00	0.00	0.00	0.00	0.00	0.00	0.00	0.00	0.00	0.00
Brain stem	Max < 15 Gy	3.09	3.09	3.19	3.10	3.09	3.10	3.10	3.09	0.00	3.19	3.24
Left eye	V8 Gy < 0.2 cc	0.00	0.00	0.00	0.00	0.00	0.00	0.00	0.00	0.00	0.00	0.00
Left eye	Max < 10 Gy	0.49	0.49	0.50	0.49	0.49	0.50	0.49	0.49	0.00	0.50	2.04
Left optic nerve	V8 Gy < 0.2 cc	0.00	0.00	0.00	0.00	0.00	0.00	0.00	0.00	0.00	0.00	0.00
Left optic nerve	Max < 10 Gy	1.27	1.22	1.30	1.27	1.29	1.19	1.39	1.19	− 6.30	1.39	9.45
Optic chiasm	V8 Gy < 0.2 cc	0.00	0.00	0.00	0.00	0.00	0.00	0.00	0.00	0.00	0.00	0.00
Optic chiasm	Max < 10 Gy	1.81	1.72	1.83	1.82	1.73	1.75	1.86	1.72	− 4.97	1.86	2.76
Right eye	V8 Gy < 0.2 cc	0.00	0.00	0.00	0.00	0.00	0.00	0.00	0.00	0.00	0.00	0.00
Right eye	Max < 10 Gy	0.52	0.49	0.56	0.50	0.53	0.51	0.51	0.49	− 5.77	0.56	7.69
Right optic nerve	V8 Gy < 0.2 cc	0.00	0.00	0.00	0.00	0.00	0.00	0.00	0.00	0.00	0.00	0.00
Right optic nerve	Max < 10 Gy	1.44	1.44	1.42	1.43	1.43	1.38	1.50	1.38	− 4.17	1.50	4.17
Skin	V23 Gy < 10 cc	0.00	0.00	0.00	0.00	0.00	0.00	0.00	0.00	0.00	0.00	0.00
Skin	Max < 26 Gy	6.15	6.26	5.85	6.33	5.79	6.06	6.15	5.79	− 5.85	6.33	2.93

Calculations were made using the Eclipse TPS

**Fig. 3:**
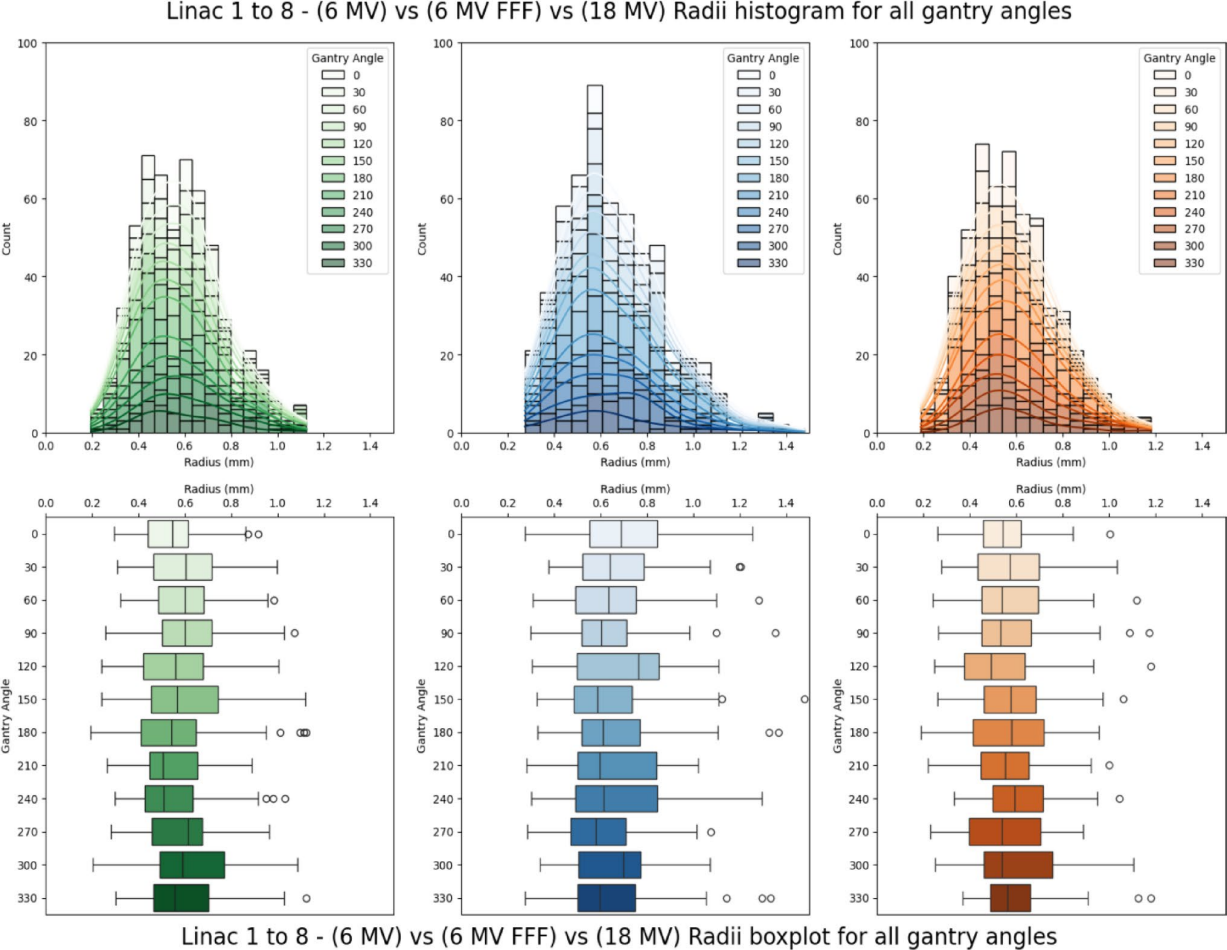
Distribution of the mean radiation isocentre radii variability across linacs 1–8

The results obtained from the extraction of radiation isocentre sizes from were binned and depicted as a histogram with counts in Fig. 3. This summary facilitates the analysis and visualisation of the distribution and contribution of different radiation isocentre sizes across various gantry angles.

**Fig. 4:**
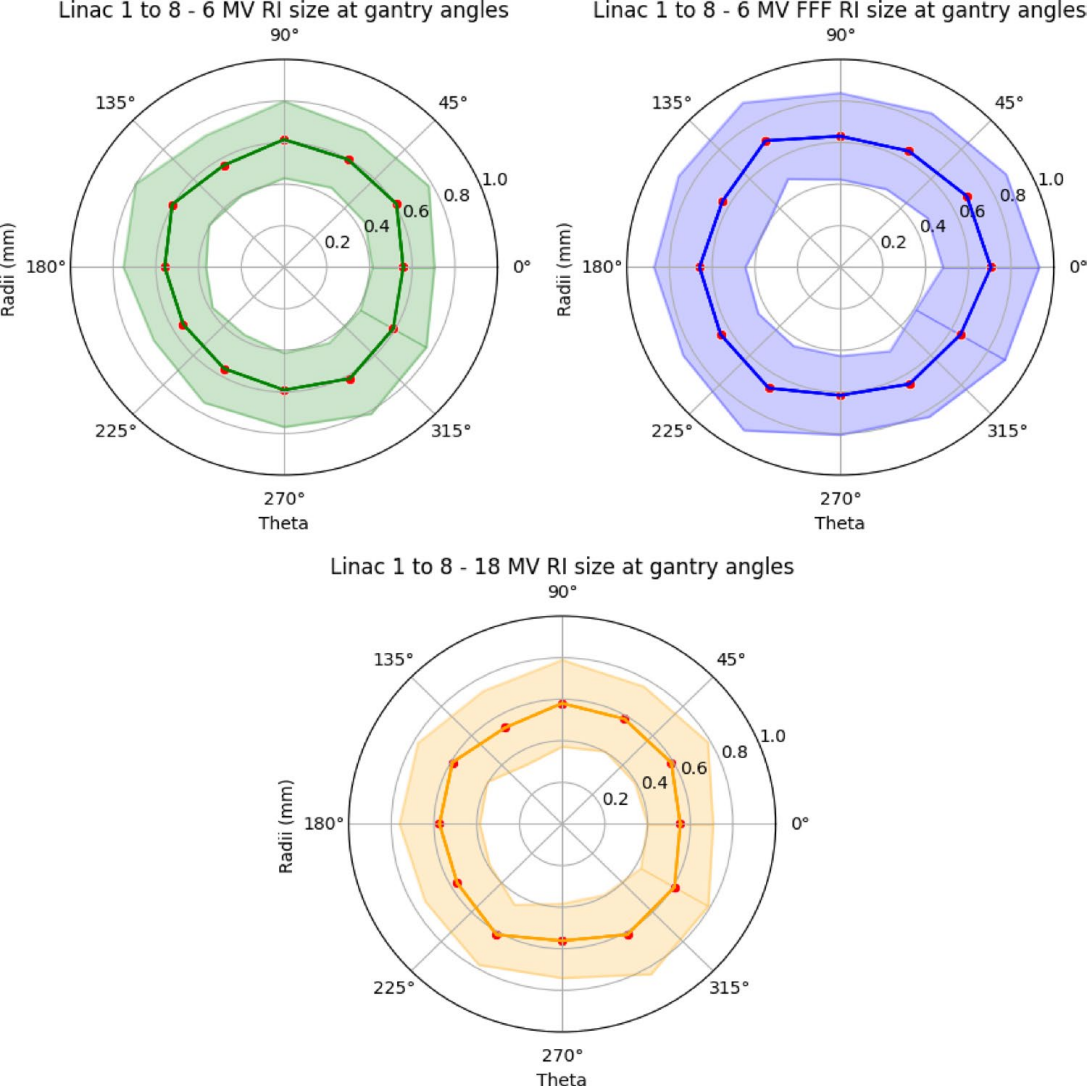
Mean radiation isocentre radii across the entire treatment arc with their corresponding standard deviations. Measurements were taken in gantry rotation steps of thirty-degree intervals

**Fig. 5:**
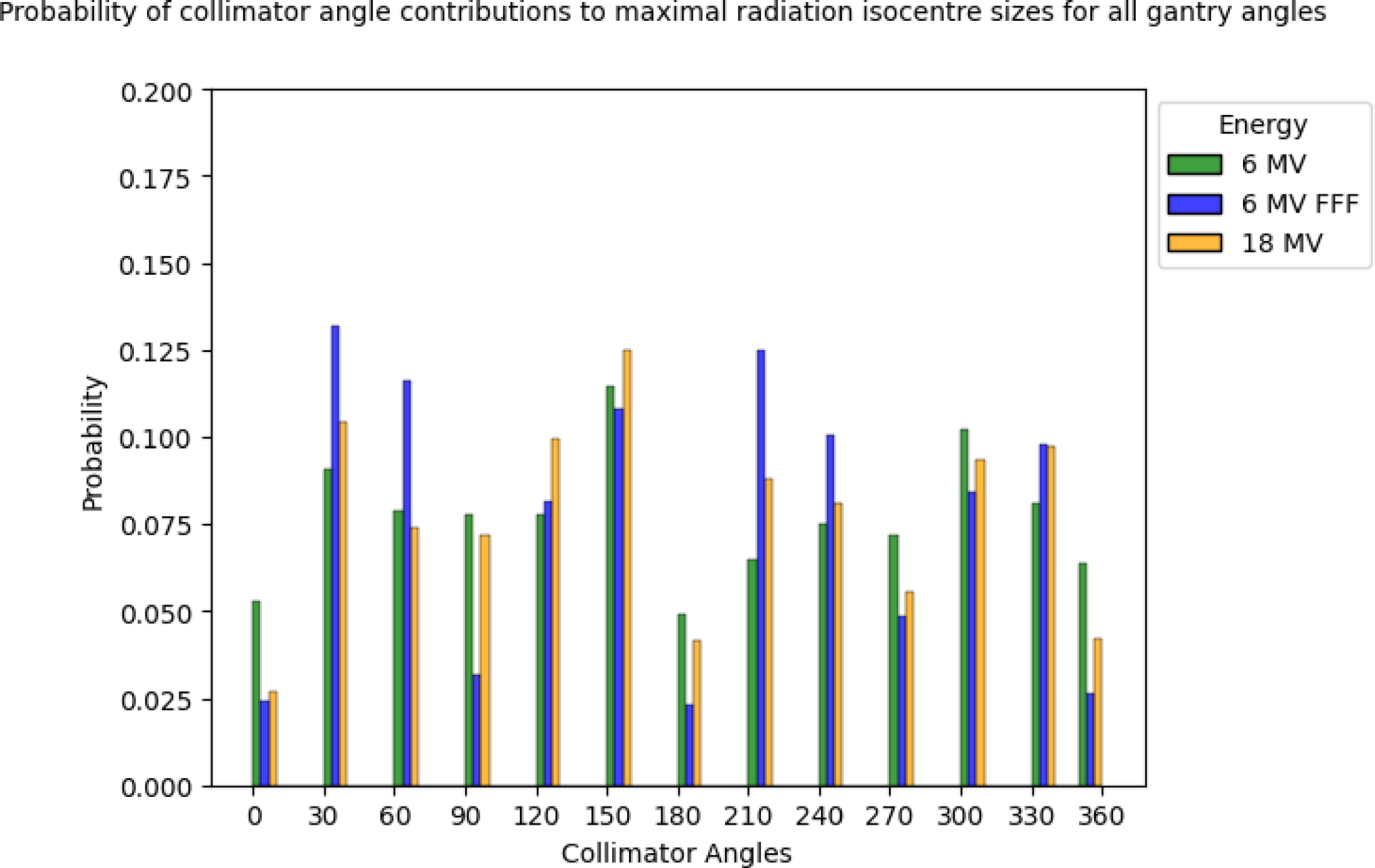
Probability distribution of each collimator angle contribution towards the largest radiation isocentre sizes across the entire treatment arc. Measurements were taken in collimator rotation steps of thirty-degree intervals at each gantry rotation angle of thirty-degrees across the entire treatment arc

The mean spatial and standard deviation values for radiation isocentres at various gantry angle configurations were summarised and visualised in Fig. 4. Meanwhile, Fig. 5 outlines the probability density of each collimator angle, highlighting their contributions to the largest radiation isocentre variabilities, separation by energies and modalities.

## Discussion

### Statistical significance

#### Shapiro–Wilk test interpretation (Table 2)

*Test statistic* In this instance, the test statistic measures 0.98, 0.96, and 0.98 respectively for 6 MV, 6 MV FFF, and 18 MV energies, all denoted as W. W serves as an indicator of how closely the data resembles a normal distribution, spanning from 0 to 1. Values approaching 1 signify a strong re-semblance to a normal distribution, while those significantly below 1 suggest deviations from the norm. It is worth noting that the standard alpha level is set at 0.05, indicating that any *p* value below this threshold would lead to the rejection of the null hypothesis, which assumes the dataset adheres to a normal distribution.

*P value* The associated *p* value for the test statistic is exceedingly small for all three energy modalities, approximately 1.20 × 10^−7^, 9.65 × 10^−11^, and 4.42 × 10^−8^ for 6 MV, 6 MV FFF, and 18 MV respectively. The *p* value quantifies the likelihood of observing the data, or more extreme data, under the assumption of a normal distribution. In this scenario, the small *p* value indicates a substantial departure from a normal distribution. Simply put, the data's alignment with normality is highly improbable.

*Interpretation* Drawing from the results of the Shapiro–Wilk test, with a test statistic of 0.98 and a remarkably small *p* value of about 1.2 × 10^−7^, it is reasonable to conclude that the data deviates significantly from normality. This departure being statistically significant, provides strong evidence to reject the assumption of normality, suggesting that non-parametric statistical tests would be more appropriate moving forward.

#### Kruskal–Wallis test interpretation (Table 3)

*Test statistic* The test statistic is a measure of the variation between groups compared to the variation with-in groups. In this case, the test statistic is 44.41. A larger test statistic indicates that there are significant differences between the groups.

*P value* The *p* value is associated with the test statistic and reflects the probability of observing the data, or data more extreme, if there were no real differences among the groups. In this case, the *p* value is extremely small, approximately 2.27 × 10^−10^. This very small *p* value suggests that it is highly unlikely to observe the data if there were no significant differences among the groups.

*Interpretation* Based on the Kruskal–Wallis test, with a test statistic of 44.41 and an extremely small *p* value of approximately 2.27 × 10^−10^, the conclusion would be that there are significant differences among the groups being compared. The test statistic being relatively large, and the very small *p* value indicate that the differences among the groups are highly statistically significant. Therefore, there is strong evidence to reject the null hypothesis of there being no significant differences among the groups, which implies that at least one group is different from the others.

#### Dunn’s test interpretation (Table 4)

The results shown in Table 4 present the *p* values obtained from the Dunn’s test, a post hoc test for pairwise comparisons following conducting the Kruskal–Wallis test. The resulting table matrix shows the *p* values for pairwise comparisons between three designated groups (labelled as 6 MV, 6 MV FFF, and 18 MV).

The diagonal elements (where the row number matches the column number) consistently always equals to 1. This aligns with expectations since comparing a group to itself should yield a *p* value of 1 because of zero differences between identical groups.

The off-diagonal elements represent the *p* values for pairwise comparisons between different groups. For example, the *p* value at row one, column two (6.43 × 10^−8^) signifies the importance of the distinction between 6 and 6 MV FFF.

To interpret these *p* values, a comparison is generally made with respect to the chosen significance level (alpha, often set at 0.05). Some common interpretations include: a *p* value less than the chosen alpha suggests a statistically significant difference between the two groups, while a *p* value greater than the chosen alpha implies statistically significant difference between the two groups.

The *p* value of 6.43 × 10^−8^ for 6 MV vs 6 MV FFF indicates a significant difference between these two groups. Additionally, the *p* value of 8.64 × 10^−9^ for 6 MV FFF vs. 18 MV also suggests a significant difference. All other comparisons, including within-group comparisons (diagonal elements), exhibit *p* values equal to 1, indicating no significant differences.

The results suggest significant distinctions between 6 and 6 MV FFF, as well as between 6 MV FFF and 18 MV. This implies that the beam energies had no significant impact on the deviations of the radii distribution, while the choice between flattened or FFF modalities significantly influenced the radii distribution. This warrants further investigation as SRS/SABR treatments are typically performed with FFF beams. This increased instability may result in coincidences and variability exceeding the tolerance levels within 1 mm during these stereotactic modalities.

### Repeatability and reproducibility

Intra and interday measurements were taken across all the linacs for each gantry and collimator angle. This was to ensure that the images acquired as part of the study were repeatable and reproducible. Table 5 is a summary of the images taken across the eight linacs.

### Mean radiation isocentre variability

According to a comprehensive study conducted by Rowshanfarzad et al*.* across Elekta, Varian, and Siemens linacs [22, 36–45], it was established that the substantial weight of radiation shielding, beam generation and shaping systems, and other gantry head components under the influence of gravity results in deviations from the ideal circular gantry rotation pattern. Rotation of the treatment beam around the patient is a common feature in radiation therapy. However, it is well-known that gantry wobbling during arc delivery adversely affects treatment spatial accuracy [36, 44, 46], particularly in arc delivery modalities such as intensity-modulated radiation therapy (IMRT), volumetric modulated arc therapy (VMAT) and stereotactic techniques like SRS/SABR, which involve highly modulated treatments with steep dose gradients [10, 47].

Collimator misalignments also occur during arc deliveries, attributed to gravity, along with the eccentricity of gantry rotation, introduce uncertainties in field shapes concerning the planned treatment fields. These factors are not initially considered in the planning systems. Gravity also induces sagging in the beam collimation system, and gantry rotation during treatment delivery can lead to additional systematic shifts in the MLC positions. These factors must be independently examined to establish tolerance limits for the MLC bank assembly positions [36, 44, 46, 48–52].

The beam focal spot, assumed to be a point source of MV X-ray generation [37, 38, 45], could be another factor contributing to the variability of the radiation isocentre. In the paper by Slama et al*.* [45], it was found that the focal spot position (FSP), when quantified, was shown to deviate from the CAX by up to 0.386 mm during gantry rotation, with it taking up to three seconds after the start of the treatment before the mean position stabilised to within 0.01 mm. This phenomenon illustrates the limitations of the electron beam steering system and the fine-tuning of the electron beam steering system could be considered following any radiation isocentre measurements.

In a study conducted by Riis et al*.* [38] eight Elekta linacs were investigated with 6 MV, 18 MV, and 6 MV FFF beams. The largest range in the FSP was found for 6 MV FFF. The FSP of one linac, retrofitted with 6 MV FFF, displayed substantial differences in FSP compared to 6 MV FFF beams on other linacs, which were all less than 0.50 mm and 0.25 mm in the lateral and longitudinal directions, respectively. Minor variations in FSP may be attributed to adjustments in beam production parameters, replacement of parts in the beam delivery system, and the wear and tear of various linac parts, including the magnetron and gun filament.

In summary, these discrepancies, which are not considered during treatment planning, necessitate consideration during plan verification measurements. Furthermore, the rotation of the linac can impact gantry-mounted accessories, such as the EPID. Given the increasing use of EPIDs in various aspects of modern radiotherapy, including dosimetry verification and real-time tumour tracking, it is essential to characterize and compensate for the mechanical imperfections within linacs as it directly impacts the position and quality of the radiation beams during treatment of patients [39–41, 43, 53].

Current TG-198 [54] guidelines only necessitate the checking of radiation and mechanical isocentre positional coincidence annually, and the recommended gantry angles are to be in steps of either 30° or 45°. This study was conducted using gantry angles of 30°. However, in addition to gantry variation, this study also included collimator angle selections at each gantry checkpoint, which are not required as part of TG-198 QA protocols, in 30° intervals.

The mean radius for the 6 MV was approximately 0.58 mm with a standard deviation of 0.18 mm. For the 6 MV FFF beam, the values were approximately 0.66 mm with a standard deviation of 0.21 mm. Finally for the 18 MV beam, the mean radius came to be approximately 0.58 mm with a standard deviation of 0.19 mm.

The Winston-Lutz test [16, 18, 49, 55] is primarily focused on ascertaining the alignment and positional coincidence of both the radiation and mechanical isocentres. The positional coincidence of the radiation and mechanical isocentres have been proven to have reached values less than 0.2 mm using this method. However, there may be cases where the coincidence range may exceed that at different sampled angles.

The results presented in this paper suggests that the radiation isocentre tolerance limit of 1 mm has potentially been violated as any positional coincidence deviation greater than a value of 0.34 mm, in the case of a 6 FFF beam, would cause a potential failure in the allowable tolerance levels for SRS and SABR treatments [54, 56].

Given the high doses involved and the presence of sensitive OAR in close proximity to treatment areas when stereotactic techniques are deployed [13–17], it seems reasonable to implement a more thorough QA procedure. More effort should be made in improving not only the number of angles sampled during QA procedures, but also in the creation of novel techniques to speed up the imaging process and potentially achieve end to end control point adjustments.

### Collimator contribution statistics

It can be seen from Fig. 5 that a cyclical pattern emerges, revealing the occurrence of maximum radiation isocentre sizes at different collimator angles. This pattern exhibits characteristics akin to a periodic function, resembling a combination of modulus sine and cosine curves as it oscillates with a 180° period. More specifically, the trough (local and global minimums) of this behaviour occurs at cardinal angles, namely 0°, 90°, 180°, and 270°.

Currently, the recommendations in TG-198 [49, 54], which proposed the use of eight images taken at four cardinal gantry angles, with two opposing collimator angles to minimise differences between mechanical and radiation isocentre localisation to within 0.2 mm. The result of this study challenges this notion. Results of this study suggests that the variability taken using the recommended cardinal angles presents the lowest probability of the largest radiation isocentre coincidence and variability being captured as part of the commissioning and QA procedure.

To better characterise isocentre variability, it is recommended to include measurements at angles where local and global maxima were observed in this study (30°, 60°, 120°, 150°, 210°, 240°, 300°, and 330°). These may differ slightly between machines, so testing a broader set of intermediate angles during commissioning and annual QA, as is consistent with current practice, would help identify machine-specific peak deviations. Continued monitoring over time could reveal whether new reference angles should be adopted and whether QA frequency needs to be adjusted based on observed trends. For stereotactic and arc-based treatments, however, assessing the full treatment arc remains the most robust approach.

### Clinical impact on SRS

Radiation Oncologists (RO) review dosimetric data presented on the TPS to determine whether a proposed treatment plan is suitable before administering. There is a recognition by clinicians that such radiotherapy plans are predictive and there are uncertainties that will ultimately impact the actual absorbed dose. Such discrepancies in absorbed dose versus planned dose have implications for both tumour control and radiotherapy related toxicity [1–4, 8, 9, 57].

In conventionally fractionated radiotherapy treatment courses, minor random errors are expected to balance out over the total treatment course with minimal clinical impact. Often these plans are also accompanied by suitable clinical target volume (CTV) to planning target volume (PTV) geometrical expansions to offset both known and unknown errors [3, 4] as well as inherent uncertainties. There is a further element of safety in the low dose per fraction schedules. However, when using a limited number (or single as in the case or SRS) of large dose(s) per fraction and very small expansions from gross tumour volumes (GTV) or CTV to PTV margin as is practiced in SRS/SABR, where tumour targets are often intimately associated with critical structures, discrepancies between planned and absorbed dose can have greater consequences. These cases may sometimes have 0 mm expansions to PTV and the dose gradients typically much steeper than conventionally fractionated schedules [16–18, 22].

SRS/SABR protocols typically include dose conformity indices, dose gradient indices and minimum target dose which serve as surrogates of quality to ensure that tumouricidal dose aligns acceptably to the target with a very rapid fall-off such that non-target tissue aligns to minimally toxic doses. Deviations in such ideal criteria may still be acceptable and are often necessary depending on the target locus and clinical context. Hence, in a SRS/SABR scenario, if the isocentre error is systematic rather than random, as appears the case in this research, and the clinical impact of a geometric error in isocentre is high secondary to rapid dose gradients, correcting the discrepancy may have greater significance. [1, 2, 8, 58, 59].

In the clinic, many OAR behave as late reacting tissues with a low alpha/beta ratio. A higher absorbed dose than predicted is met with a magnified biological impact as it is not just the total dose received but also the dose per fraction which contributes to the risk of toxicity. Ultimately an exponential component to cell death rather than solely linear as predicted by the linear quadratic model. Spinal cord, optic chiasm, optic nerve, brainstem, duodenum, trachea and major bronchi are examples of late reacting tissues where failure to predict absorbed dose hot spots could lead to life altering or even lethal sequelae. There is a more linear relationship with most tumour targets which are characterised as early reacting tissues (high alpha beta ratio) [1, 2, 8, 11, 59, 60] although prostate cancer and some benign tumours are exceptions. A lower than predicted absorbed dose to the tumour target would be anticipated to be less effective in controlling the disease.

The clinical goals summary presented in Tables 6 and 7 demonstrate that when the error in isocentre is taken into account and the appropriate adjustment made, there are changes in dose distribution from the original plan, impacting OAR and target parameters. Ultimately the clinical relevance will depend on the case [61]. From these examples there is a consistent failure to meet the set clinical goals beyond the PTV, which is anticipated across both single and multi-lesion scenarios. This discrepancy can be explained by considering additional PTV margins from an inverse perspective. The outermost PTV, PTV_2 mm, serves as a proxy for plans where the RO opted for 0 mm margin to PTV expansions. Similarly, PTV_1 mm represents plans with a 1 mm margin to PTV, while the GTV corresponds to plans with a 2 mm margin. Both perspectives draw the conclusions of the importance of including margins with magnitudes that can absorb uncertainties. It is apparent that these revised plans would not meet all prescription goals and as a result, the treating clinician would likely reject due to concerns for treatment efficacy. 

The analysis of OAR in the two brain SRS examples provided reveals that there were no clinically relevant dosimetric changes with no breaching of established dose threshold criteria. However, it is notable that the magnitude of percentage maximum change is considerable in certain instances (27.78% in one instance) which could raise concern in other examples where target geography is more closely approximated to a critical structure.

## Conclusion

With the emergence of new treatment methods in modern radiotherapy and the rapid proliferation of SABR and SRS which involve delivering high radiotherapy doses in a very precise, focused nature, reducing any margin for error is critical. This is particularly critical when treatment targets are in close proximity to highly sensitive OAR.

Whilst the two brain stereotactic plan examples provided in this manuscript did not identify absolute dosimetric differences on OAR constraints of clinical significance, the maximal percentage differences in dose in some examples were large. These differences were primarily due to the relatively small absolute doses involved by those structures. It is conceivable that other scenarios, particularly when the target lies adjacent to a critical structure, the dosimetric changes could be more likely to produce a clinical impact. Therefore, it is essential to ensure a comprehensive understanding of the concept of the radiation isocentre, and to incorporate a thorough characterisation of radiation isocentre variability throughout the entire treatment arc into the treatment planning process.

In addition to the current standard commissioning and QA protocols outlined in TG-142 [56] and TG-198, it is advisable to update current protocols for measuring radiation isocentre variability. We propose enhancing protocols by incorporating a hybrid approach that combines traditional Starshot methods with a Winston-Lutz-style ball bearing setup. This allows evaluation of both the positional deviation from the mechanical isocentre and the overall radiation isocentre variability across a broader range of gantry and collimator angles. By capturing these data during commissioning and ongoing QA, institutions can better define machine-specific reference angles and tailor QA frequency according to observed mechanical trends and temporal changes. This recommendation complements existing protocols and aims to provide a more complete characterisation of linac specific radiation isocentre behaviour.

Following the current recommendations provided in TG-198, the assessment of radiation isocentricity for each axis (collimator, gantry, and treatment couch) is commonly conducted using the Starshot technique. This technique checks the positional alignment of the radiation and mechanical isocentres. Specifically, the intersection of the lines should fall within a specified tolerance circle for the machine [16, 54, 56, 62]. Although the Winston-Lutz test [16, 18, 22, 49, 63] has been a standard QA tool since its development in 1988, it remains widely adopted for its simplicity and ability to assess isocentre accuracy across all three axes. This study extends its application by evaluating the full spatial variability of the isocentre, not just positional deviation, across multiple gantry and collimator angles. This variability can change over time and may be linac specific, suggesting that QA frequency should be based on machine-specific baselines established during commissioning and adjusted according to observed temporal trends within the clinic.

It is advisable, from the results of this study, to measure radiation isocentre variability at different gantry and collimator angles, in 30° increments, to capture the linac specific true geometric variation across the entire treatment arc. The resulting mean radiation isocentre variation should be added to the positional deviation measured by the Winston-Lutz test and incorporated into the TPS, providing a more accurate representation of the radiation isocentre size deviation from the mechanical isocentre position. This precision is particularly vital as the current allowable threshold deviation for SABR, and SRS treatment techniques is set at 1 mm.

This study proposes a linac specific determination of optimal gantry and collimator angles to be determined at commissioning using both the Starshot and Winston-Lutz test to capture both the positional deviation and the variability of the radiation isocentre. This baseline can then be verified through regular QA tests as recommended by TG-198 to track the degradation of the machine over time or changes due to any mechanical services. Future work would be to develop techniques to assess off-axis beams such as those used for multi-lesion brain SRS.

## Appendix 1

### Program structure

**Table:**

ALGORITHM 1: ALGORITHM TO EXTRACT RADIATION ISOCENTRE POSITION AND SIZE		
1	for *dicom images* **in** gantry angles:	
2		read *dicom images* using **pydicom**
3		apply **loadEPID** ← pixel offset dependent on linac manufacturers (e.g. Varian, Elekta, Siemens)
4		apply **median** using **opencv** ← median filter for edge preservation noise reduction
5		apply **canny** using **opencv** ← detecting field edges
6		index *edge positions* ← row and column indexes stored as a paired list
7		apply **scandirection** to *edge positions* ← determines a vertical or horizontal scan direction
8		apply **pixelposition** ← determines the pixel position of the left and right edges per edge pair
9		apply **cubicregression** ← determines the subpixel position of the left and right edges per edge pair
10		apply **linearregression** ← determines the CAX position of each EPID field
11		apply **collimatorangle** ← determines the collimator angle for each field using the arctangent function
12		apply **NchooseK** ← finds all the possible set of three combinations of the Starshot field lines
13		apply **heron** ← applies heron’s formula to each set of combinations to determine both the size and position of the radiation isocentre
14		output *final combinations* ← list of all possible combinations and the corresponding radiation sizes and positions
15	**end**	
